# ULK1 mediated autophagy in airway cells during *Aspergillus* infection

**DOI:** 10.3389/fmicb.2026.1756294

**Published:** 2026-03-17

**Authors:** Fangyan Chen, Rui Zhao, Yuqing Sun, Yangxuan Lin, Xiao Cui, Jingya Zhao, Yingsong Hu, Zelei Wang, Dingchen Li, Mandong Hu, Li Han

**Affiliations:** 1 Department for Disinfection and Infection Control, Chinese PLA Center for Disease Control and Prevention, Beijing, China; 2 Department of Respiratory and Critical Care Medicine, Beijing Youan Hospital, Capital Medical University, Beijing, China; 3 National Center of Biomedical Analysis, Beijing, China

**Keywords:** *Aspergillus fumigatus*, autophagy, bronchial epithelial cell, complement receptor 3, LC3-associatedautophagy, ULK1

## Abstract

**Introduction:**

*Aspergillus fumigatus* is a major airborne fungal pathogen that causes invasive aspergillosis in immunocompromised individuals. This study aims to elucidate the autophagic mechanisms activated following the internalization of *A. fumigatus* conidia in human bronchial epithelial cells.

**Methods:**

Specifically, we investigated the role of ULK1 and the autophagic processes in Beas2B cells upon *A. fumigatus* conidia internalization. The Beas2B cell line was used to assess the protein expression of ULK1, phosphorylated ULK1, and LC3-I/II via Western blotting. Autophagosome structures were examined using transmission electron microscopy. Gene silencing of ULK1 using siRNA and pharmacological inhibition with SBI-0206965 were performed. Secreted inflammatory cytokines were quantified using specific immunoassays.

**Results:**

*A. fumigatus* conidia induced a time- and dose-dependent conversion of LC3-I to LC3-II, indicating autophagic activation resembling LC3-associated phagocytosis (LAP). ULK1 expression significantly increased post-infection, whereas genetic silencing of ULK1 reduced LC3-II conversion. Notably, common fungal polysaccharides and Dectin-1 did not influence this process, but the loss of complement receptor 3 (CR3) elevated both basal and conidia-induced autophagy, correlating with increased AMPK expression.

**Discussion:**

This study reveals a novel ULK1-dependent autophagic response similar to LAP during *A. fumigatus* internalization, highlighting potential therapeutic targets for managing invasive aspergillosis in immunocompromised patients.

## Introduction

*Aspergillus fumigatus* (*A. fumigatus*) is a WHO priority fungal pathogen (Jia et al., 2024) and a major cause of invasive pPulmonary aspergillosis (IPA), a severe infection increasingly problematic due to rising susceptible populations (e.g., immunocompromised patients, those with COPD, and ICU patients) and azole resistance (Fekkar et al., 2021). IPA affects over 2.1 million people annually and results in high mortality (1.8 million deaths) (Denning, 2024), the infection is initiated when inhaled conidia lodge in the airways (Tischler and Hohl, 2019).

Traditionally, research on host defense focused on immune cells such as macrophages and neutrophils, which utilize LC3-associated phagocytosis (LAP), a Rubicon-dependent, noncanonical autophagy pathway characterized by single-membrane vesicles to clear fungi (Espinosa et al., 2017; Martinez et al., 2015). However, airway epithelial cells, which constitute the first line of defense, also play a crucial role (Han et al., 2021). It remains unclear whether epithelial cells utilize LAP or a similar strategy to defend against *A. fumigatus*.

Autophagy, a vital process for cellular homeostasis and stress response, includes classical autophagy (ULK1-dependent, double-membrane autophagosomes) and noncanonical LAP (ULK1-independent, NOX2/Rubicon-dependent, single-membrane vesicles), both pathway are marked by LC3-II lipidation (Jia et al., 2024; Forn-Cuní et al., 2023; Chen et al., 2024; Florey et al., 2011). LAP is triggered by specific immune receptors such as Dectin-1, TLRs, and CR3 (Oikonomou et al., 2018). Currently, the mechanism by which epithelial cells select between these autophagy pathways in response to fungal infection remains unknown.

Lung epithelial cells act as barriers and aid in conidia clearance (Feys et al., 2022), and evidence suggests that *A. fumigatus* induces autophagy during epithelial internalization (Cui et al., 2023; Toor et al., 2018). Complement receptor 3 (CR3) mediates phagocytosis via complement or direct fungal recognition (Fernández et al., 2022) and is linked to LAP in macrophages (Herb et al., 2018; Gluschko et al., 2018). Autophagy regulation involves AMPK (activating ULK1) and mTOR (inhibiting ULK1) (Florey et al., 2011). Key questions persist regarding *A. fumigatus*-induced autophagy in epithelial cells: Is it induced? What is CR3’s impact on this process and the AMPK/mTOR pathway? And what is ULK1’s specific function?

In this study, we investigated the autophagic response to *A. fumigatus* conidia in human Beas2B epithelial cells. We uncovered a novel LAP-like autophagy pathway that, distinct from canonical LAP, is mediated by ULK1. We further demonstrate CR3’s role in modulating this pathway via the negative regulation of AMPK signaling. Our findings identify a previously unknown LAP-like mechanism in airway epithelial cells, bridging a knowledge gap in fungal-host interactions, advancing the mechanistic understanding of IPA, and highlighting potential therapeutic targets for modulating host autophagy against fungal infections.

## Materials and methods

### *Aspergillus fumigatus* strain and cell line

The *A. fumigatus* wild type strain B5233 was a gift from Dr. KJ. Kwon-Chung (National Institute of Health, Bethesda, Maryland) (Sugui et al., 2007). The *A. fumigatus* ATCC13073 constitutively expressing green fluorescent protein was generously provided by Dr. Margo Moore (Simon Fraser University, Burnaby, BC, Canada) (Han et al., 2021). The mCherry-labeled *A. fumigatus* CEA17*Δku80* strain was constructed by our team’s laboratory. The *A. fumigatus* conidia was propagated on Sabouraud dextrose agar (10 g/L peptone, 10 g/L glucose, 15 g/L agar, pH 6.0) for 5–8 days at 37 °C and prepared as described previously (Han et al., 2021). The conidia were passed through 8 layers of sterile gauze to remove hyphal fragments, then washed twice and enumerated using a hemacytometer. Heat inactivation was performed at 121 °C for 15 min in an autoclave.

The human bronchial epithelial cell line Beas-2B and type II human alveolar epithelial cell line A549 were provided by Yuanjing Biotechnology Co., Ltd. (Guangzhou, China, Cat. No. UBISCE211210JW3 and UBISCE211210JW2). The parental cell line was originally sourced from the American Type Culture Collection (ATCC, Manassas, VA, USA) and cultured in RPMI-1640 (GIBCO, Germany) supplemented with 10% heat-inactivated foetal calf serum, 100 U/mL streptomycin, and 100 U/mL penicillin at 37 °C in an atmosphere of 5% CO_2_.

### Transmission electron microscopy (TEM)

Beas2B and A549 cells were fixed (2.5% glutaraldehyde, followed by 1% osmium tetroxide), dehydrated through a graded ethanol series, embedded in epoxy resin, and ultrathin-sectioned using a Leica EM uc7 ultramicrotome. Sections were stained, mounted on copper grids, and imaged using a Hitachi HT7700 or HT7800 TEM (80–120 kV) following standard procedures.

### Chemical reagents, antibodies, siRNAs and plasmids

LC3 Rabbit Polyclonal antibody(#14600-1-AP, 1:1000), Rubicon Rabbit Polyclonal Antibody (#21444-1-AP, 1:1000), GAPDH Polyclonal antibody(#10494-1-AP, 1:2000) were purchased from proteintech, Phospho-UKL1(Ser555) Rabbit monoclonal antibody (#5869, 1:500), Phospho-UKL1(Ser757) Rabbit monoclonal antibody (#6888, 1:500), ULK1 rabbit monoclonal antibody (#8054, 1:1000), SQSTM1/p62 antibody (#5114, 1:1000), Atg5 Rabbit monoclonal antibody (#12994, 1:1000), Dectin-1 Rabbit monoclonal antibody (#60128, 1:1000), AMPKalpha Antibody (#2532, 1:1000), Phospho-AMPKalpha (Thr172) Rabbit monoclonal antibody(#50081, 1:1000), *β*-tubulin Rabbit monoclonal antibody(#2128, 1:2000) were purchased from Cell Signaling Technology (USA). Anti-VPS34 Rabbit Polyclonal antibody (#GB13161, 1:1000) was purchased from Wuhan Seville Biotechnology (China). HPR-conjugated goat anti-mouse IgG and HRP-conjugated goat anti-rabbit IgG antibodies were obtained from ZSGB-BIO (China, 1:4000). DAPI (4′,6-diamidino-2-phenylindole) was purchased from Sigma-Aldrich (#D9542). Alexa Flour-conjugated Goat anti-Rabbit IgG secondary antibody.

was purchased from Thermo Fisher (#A32731). siRNA-ULK1 (siRNA ID: SASI_Hs02_00335869), siRNA-PI3K (siRNA ID: SASI_Hs01_00233716), siRNA-Rubicon (siRNA ID: SASI_Hs02_00346052) and siRNA universal negative control were designed and chemically synthesized by Sigma-Aldrich (USA). Plasmid encoding LC3B conjugated to enhanced green fluorescent protein (eGFP) (eGFP-LC3B) was kindly gifted by professor Yaling Xing (Academy of Military Medicine Sciences) (Han et al., 2018).

### Treatment of Beas2B cells

Beas2B (5 × 10^5^cells/well) and A549 (1 × 10^6^cells/well) cells were seeded in 6-well plates. For infection, cells were incubated in serum-free medium with *A. fumigatus* resting conidia (MOI = 10) for the indicated times at 37 °C, 5% CO_2_. For inhibition studies, cells were pre-treated with ULK1/2 inhibitor (different concentrations of SBI-0206965 in DMSO for 24 h) before infection. Uninfected and vehicle (DMSO) controls were included.

### Western blotting analysis

Cells were lysed in RIPA buffer containing protease and phosphatase inhibitors. Protein concentration was determined using a BCA assay. Equal amounts of protein were separated by SDS-PAGE and transferred to PVDF membranes. Membranes were blocked with 5% BSA for 2 h, incubated with primary antibodies at 4 °C overnight, followed by HRP-conjugated secondary antibodies at room temperature for 1 h. Signals were detected using ECL reagent, and band intensity was quantified using ImageJ and normalized to GAPDH.

### Immunofluorescence analysis

Beas2B and A549 cells on coverslips were seeded in 24-well plates at a density of 5 × 10^4^ cells/well and cultured overnight. In Beas2B cells, when the cell confluency reached 70–80%, the eGFP-LC3B plasmid was transfected into the cells using Lipofectamine 2000 according to the manufacturer’s instructions. After transfection for 48 h, the cells were treated with mCherry-labeled *A. fumigatus* CEA17*Δku80* conidia (MOI = 10) separately for 4 h, 6 h, and 8 h (0 h as the control group, without conidia infection). After incubation, cells were fixed with 4% paraformaldehyde at room temperature for 15 min, then permeabilized with 0.1% Triton X-100 for 10 min. Subsequently, the cells were incubated with DAPI working solution at room temperature in the dark for 5 min, and finally washed 3 times with PBS to remove excess dye. In A549 cells, when the cell confluency reached 70–80%, the cells were treated with *A. fumigatus* ATCC13073 conidia (MOI = 10) separately for 4 h, 6 h, and 8 h (0 h as the control group, without conidia infection). After incubation, cells were fixed with 4% paraformaldehyde at room temperature for 15 min, then permeabilized with 0.1% Triton X-100 for 10 min. The samples were blocked in 5% bovine serum albumin (BSA) at room temperature for 30 min, and incubated with primary antibodies (LC3B) at 4 °C overnight and then with Alexa Flour-conjugated secondary antibodies (red) for 1 h. Subsequently, the cells were incubated with DAPI working solution at room temperature in the dark for 5 min, and finally washed 3 times with PBS to remove excess dye. Images were acquired using an Olympus BX51 fluorescence microscope (400×).

### Internalization assay

Fungal internalization was assessed using a nystatin protection assay. After washing to remove extracellular conidia, cells were lysed (0.25% Triton X-100, 15 min). Released intracellular conidia were plated on SDA agar and incubated at 37 °C for 18 h, after which colonies were counted. Internalization was expressed as a percentage of the initial inoculum.

### Cytokine measurement

Concentrations of IL-6, IL-8, MCP-1 (Solarbio kits), IL-12, and IL-23 (R&D Systems kits) in cell culture supernatants were measured using specific ELISA kits according to manufacturer’s instructions.

### Quantitative real-time PCR

Total RNA was extracted (TRIzol) and reverse transcribed (RevertAid Kit). cDNA was quantified via qPCR using SYBR Green Master Mix (Tian Gen) with primers listed in Table 1. Cycling conditions included 40 cycles (94 °C/5 s, 60 °C/30s). Relative expression was calculated using the 2 − ΔΔCT method and normalized to GAPDH.

**Table 1:** Primers used in the study.

Name	Forward 5′–3′	Reverse 5′–3′
GAPDH	ATCCCATCACCATCTTCCAG	CCATCACGCCACAGTTTCCC
ITGAM	GGCTCTGGTAGCATCATCCC-3′	CCCAAGCAGCTGCGTTATTG-3′
β-actin	CGAGCACGGCATCGTCAC	CTGGATAGCAACGTACATGGC
ULK1	AGAACAAGACGTTGGTCCCC	AGAACAAGACGTTGGTCCCC
CD11b	GCTTTGGTGGCTTCCTTGTG	TAGTCGCACTGGTAGAGGCT
ATG5	GATGGCGGGTGAAGGTGGTT	TGCCATTTCAGTGGTGTGCC
Rubicon	GCGGGCACCTCTGAATCTTA	CCTCCACCGTCGTCTTCAAA
Vps34	GCTTAAGATCTGGAATGAATGGC	AGATCGTGGTCAGAAGGTCCA

### Statistical analysis

Data are presented as mean ± SD from three independent experiments. GraphPad Prism was used for analysis. Student’s *t*-test or one-way ANOVA with Tukey’s *post hoc* test was used to determined significance (*p* < 0.05).

## Results

### *Aspergillus fumigatus* conidia induced an increase of LC3-II in Beas2B cells

**Figure 1:**
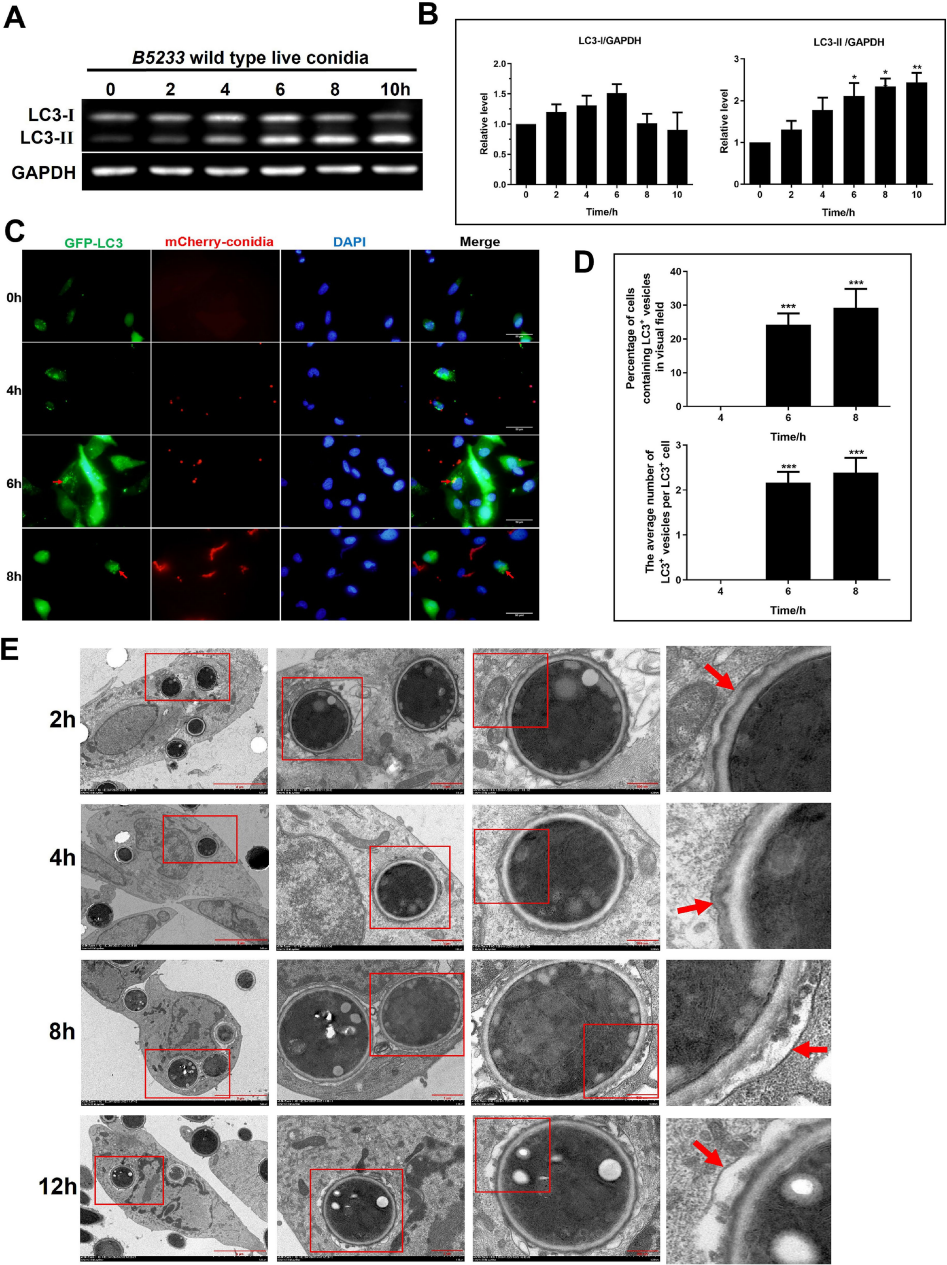
Increased LC3-II expression and monolayer membrane-coated autophagosome formation in Beas2B cells following conidia infection. **(A)** Expression of LC3-I and LC3-II in Beas2B following infected by B5233 wild-type live conidia at 0, 2, 4, 6, 8, and 10 h. **(B)** Relative expression levels of LC3-I and LC3-II at the various time points. **(C)** Fluorescence microscopy images of GFP-LC3 (green), mCherry-labeled conidia (red), and DAPI staining (blue) in Beas2B cells following infected by conidia at 0, 4, 6, and 8 h. **(D)** Quantification of cells with LC3^+^ vesicles and average number of LC3^+^ vesicles per cell at 4, 6, and 8 h. **(E)** Electron microscopy images of cellular structural changes over time at 2, 4, 8, and 12 h. The magnification progressively increases from left to right, with each subsequent column of images depicting the area enclosed within the red box of the preceding image. The red arrow in each image points to the monolayer membrane encapsulating the *A. fumigatus* spores. ^*^p < 0.05; ^**^p < 0.01; ^***^p < 0.001.

*Aspergillus fumigatus* infection progressively increased LC3-I and LC3-II levels in Beas2B cells over 0–6 h, with LC3-II significantly elevated at 6 h compared to 0 h and continuing to rise up to 10 h (Figures 1A,B). Confocal microscopy showed mCherry-conidia (red) surrounded by GFP-LC3-II (green), visualized as yellow rings (Figure 1C). At 6–8 h post-infection, 24–29% of cells contained LC3-II-encapsulated conidia, averaging approximately 2 per cell (Figure 1D). TEM revealed conidia enveloped by single-membrane vesicles from 2 to 12 h post-infection (Figure 1E), suggesting a LAP-like process rather than canonical double-membraned autophagy. Consistent results were observed in the human type II alveolar epithelial cell line A549 (Supplementary Figures S1A–D). During infection with *A. fumigatus*, intracellular LC3-I levels gradually decreased, whereas LC3-II levels increased. A small number of intracellular conidia were surrounded by yellow rings, and the membrane encasing the conidia was a single-layer membrane. Notably, the induction of LC3-II was less prominent in A549 cells than in Beas-2B cells. Analysis of conidial surface components indicated that polysaccharides, hydrophobins, and melanin were likely not the primary autophagy triggers; however, heat-sensitive components appeared essential (Supplementary Figures S2A–F).

### ULK1-dependent autophagy and LC3-II conversion in Beas2B cells during *A. fumigatus* infection

**Figure 2:**
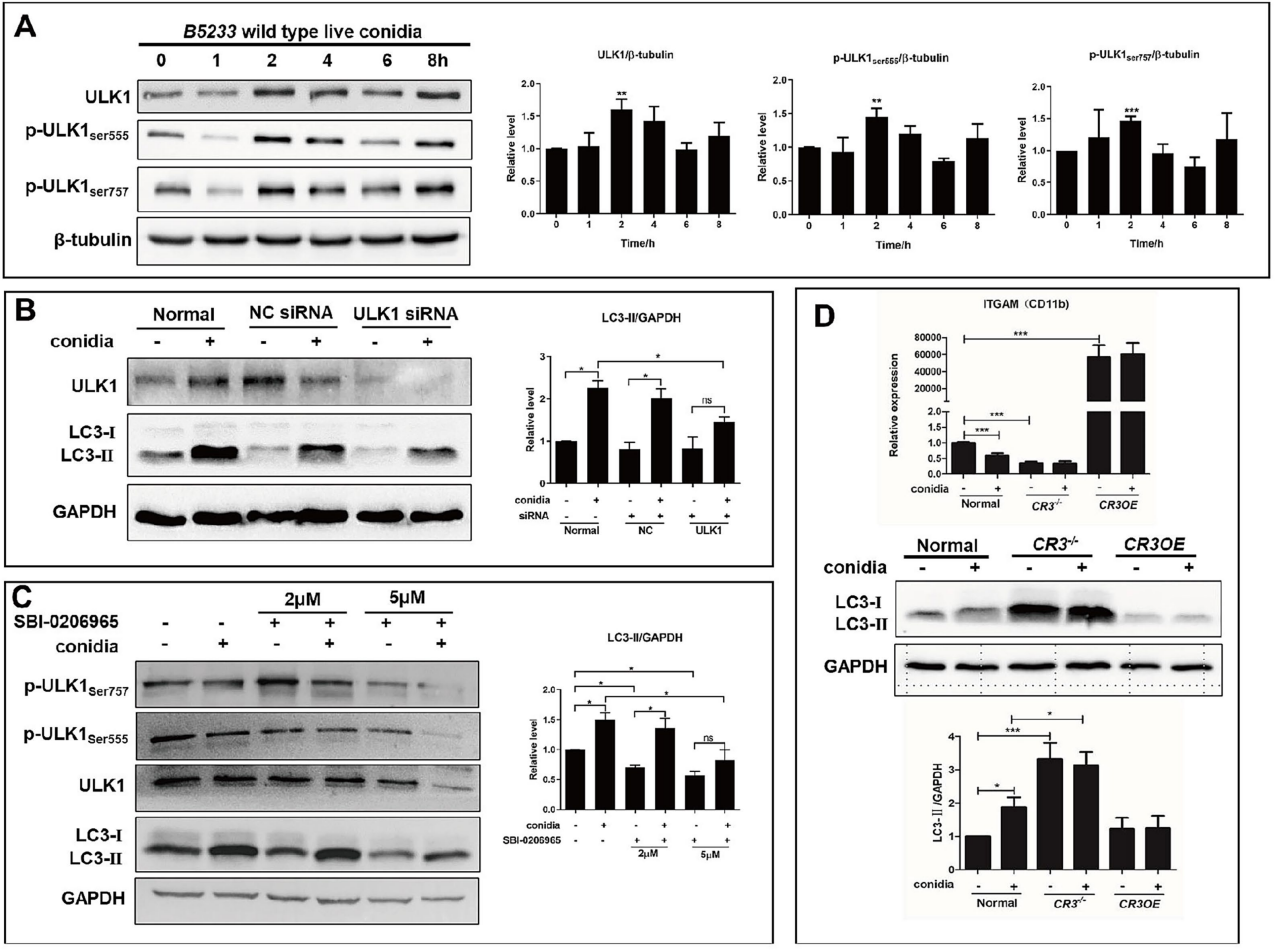
ULK1 and CR3 receptor modulated conidia-induced autophagy in Beas2B cells. **(A)** Expression levels of ULK1, p-ULK1 (Ser555), and p-ULK1 (Ser757) in Beas2B after internalization by B5233 wild-type live conidia at 0, 1, 2, 4, 6, and 8 h. **(B)** Expression levels of LC3-I and LC3-II in ULK1-silenced Beas2B cells after internalization by conidia. **(C)** Expression levels of LC3-I, LC3-II, ULK1, p-ULK1 (Ser555), and p-ULK1 (Ser757) in Beas2B cells treated with 2 μM and 5 μM SBI-0206965 inhibitor targeting ULK1/2 with conidia infection. **(D)** Expression levels of LC3-I and LC3-II in Beas2B cells with CR3 knockdown or overexpression after internalization by conidia. ^*^p < 0.05; ^**^p < 0.01; ^***^p < 0.001.

ULK1 expression and phosphorylation (Ser555, Ser757) significantly increased by 2 h post-infection (Figure 2A). Silencing ULK1 with siRNA significantly reduced *A. fumigatus*-induced LC3-II levels (Figure 2B). Pretreatment with 5 μM ULK1/2 inhibitor SBI-0206965 significantly inhibited ULK1 phosphorylation and LC3-II induction (Figure 2C). We investigated the role of CR3 (CD11b). In CR3-deficient (CR3^−/−^) Beas2B cells, basal LC3-II levels were significantly elevated, and *A. fumigatus* infection caused no further increase, unlike in wild-type cells (Figure 2D). Conversely, CR3 overexpression (CR3OE) suppressed *A. fumigatus*-induced LC3-II elevation (Figure 2D). However, this effect was less pronounced in A549 cells than in Beas2B cells (Supplementary Figures S1E,F). These results suggest that CR3 negatively regulates both basal and induced LC3-II conversion.

### AMPK might be involved in CR3-regulated ULK1 expression in Beas2B cells

**Figure 3:**
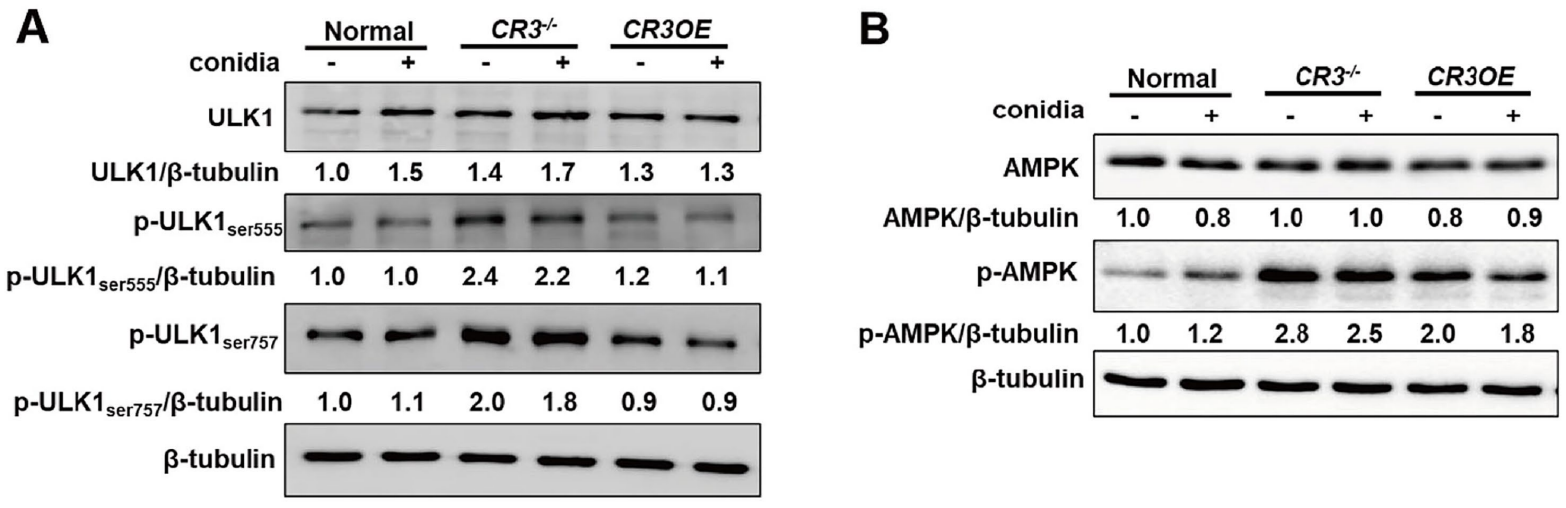
AMPK involved in CR3-regulated ULK1 expression in Beas2B cells. **(A)** Expression levels of ULK1, p-ULK1 (Ser555), and p-ULK1 (Ser757) in Beas2B cells with CR3 knockdown (CR3^−/−^) or overexpression (CR3OE) after conidia stimulation. **(B)** Expression levels of AMPK and p-AMPK in Beas2B cells with CR3^−/−^ or CR3OE after conidia stimulation. ^*^p < 0.05; ^**^p < 0.01; ^***^p < 0.001.

While *A. fumigatus* slightly increased total ULK1, its phosphorylation at Ser555 and Ser757 was enhanced in CR3^−/−^ cells, irrespective of infection, but remained unaltered in CR3OE cells compared to wild-type controls (Figure 3A). *Aspergillus fumigatus* promoted the expression and phosphorylation of AMPK, an upstream regulator of ULK1. This activation was enhanced in CR3^−/−^ cells and attenuated in CR3OE cells, regardless of infection status (Figure 3B). This indicates *A. fumigatus* activates LC3-II conversion via an AMPK/ULK1 pathway, which is negatively regulated by CR3.

### ULK1, but not Rubicon or Dectin-1, Regulated *A. fumigatus* conidia-induced autophagy in Beas2B cells

Expression of the LAP regulator Rubicon decreased significantly only at late time point (10 h post-infection), while Atg5 and VPS34 levels remained unchanged (Supplementary Figures S3A–D). Furthermore, their mRNA levels were examined in Beas2B and A549 cells. In Beas2B cells, the mRNA expression pattern was consistent with the protein level, but differed slightly from that in A549 cells (Supplementary Figure S4). During *A. fumigatus* infection, Rubicon and CD11b mRNA levels significantly decreased consistently in both cell lines, whereas Atg5, VPS34, and ULK1 mRNA levels differed between them: Beas2B cells showed a gradual significant decrease in Atg5 and VPS34 mRNA levels and a gradual increase in ULK1 mRNA levels, while A549 cells exhibited no obvious changes in Atg5 and VPS34 mRNA levels and a biphasic trend (first increase, then decrease) in ULK1 mRNA levels. p62 levels decreased significantly at 12 h (Supplementary Figures S3E,F). While intracellular ROS (necessary for canonical LAP via NOX2) peaked at 6 h (Supplementary Figure S3G), silencing Rubicon or VPS34 did *not* affect the *A. fumigatus*-induced LC3-II increase (Supplementary Figures S3H,I). Dectin-1 was expressed but unaffected by infection (Supplementary Figure S3J), and neither Dectin-1 knockout nor overexpression altered conidia-induced LC3-II levels (Supplementary Figure S3K). Thus, ULK1, but not Rubicon or Dectin-1, is the key regulator in this pathway.

### ULK1 and CR3 modulated inflammatory factor release and *A. fumigatus* internalization in Beas2B cells

**Figure 4:**
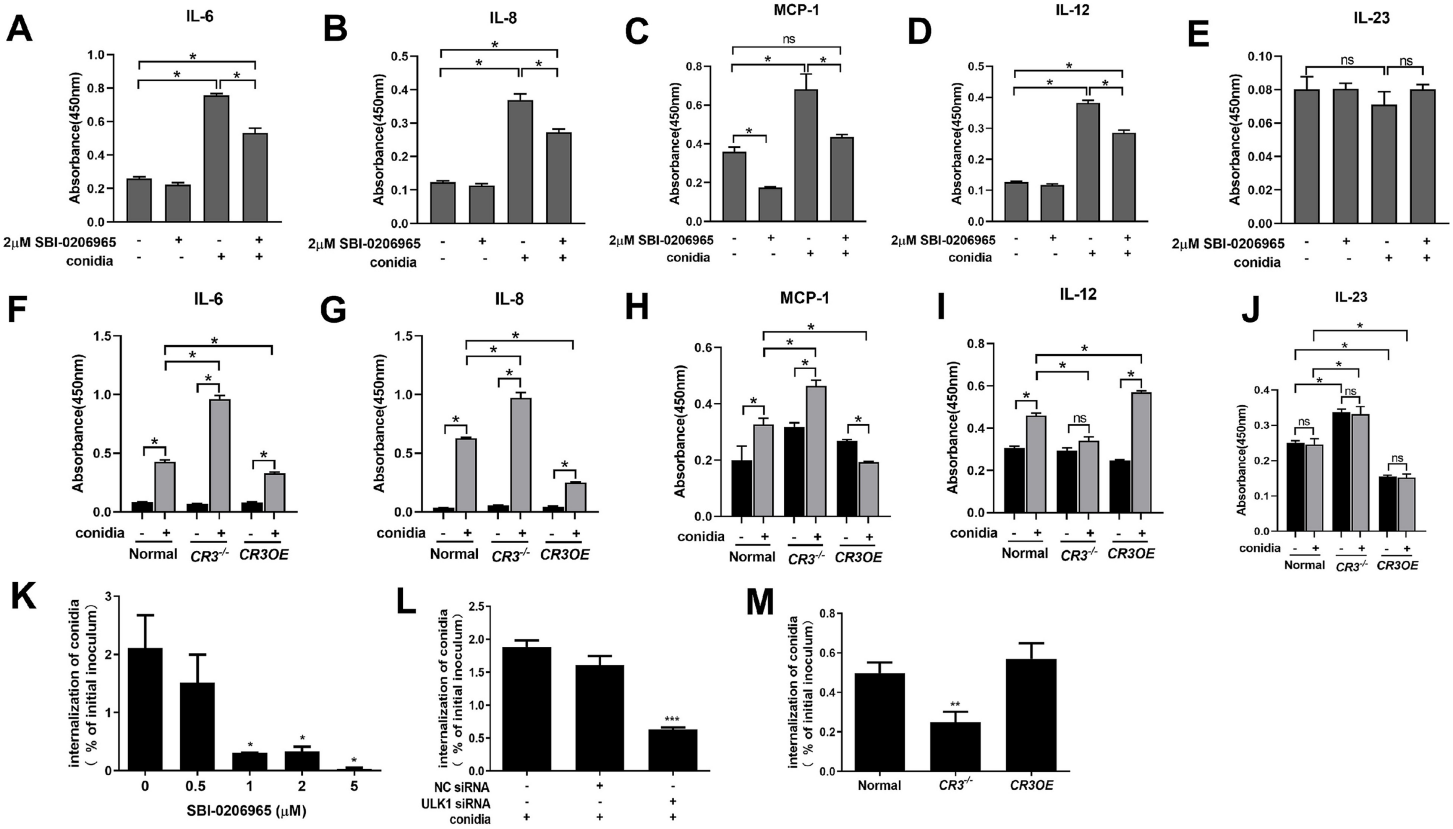
Effect of ULK1 and CR3 on the release of inflammatory factors and internalization of *A. fumigatus* into Beas2B cells. **(A–E)** Quantification of IL-6, IL-8, MCP-1, IL-12, and IL-23 levels in different treatment groups (2 μM SBI-0206965 pretreatment for 24 h and conidia infection for 12 h). **(F–J)** Quantification of IL-6, IL-8, MCP-1, IL-12, and IL-23 levels following internalization of live conidia, with CR3 knockdown or overexpression. **(K)** Quantification of conidia internalization by different concentrations of SBI-0206965 treatment. **(L)** Quantification of conidia internalization by silence RNA specific for ULK1. **(M)** Quantification of conidia internalization with CR3 knockdown or overexpression. ^*^p < 0.05; ^**^p < 0.01; ^***^p < 0.001.

Inhibiting ULK1 with SBI-0206965 significantly reduced *A. fumigatus*-induced release of IL-6, IL-8, MCP-1, and IL-12 (but not IL-23) (Figures 4A–E). CR3 loss enhanced the release of IL-6, IL-8, and MCP-1 induced by *A. fumigatus*, while inhibiting IL-12, whereas CR3 overexpression showed opposing trends (Figures 4F–I). IL-23 release was enhanced by CR3 loss and suppressed by CR3 overexpression, irrespective of the presence or absence of *A. fumigatus* (Figure 4J). Regarding fungal internalization, a key step in pathogenesis (Margalit et al., 2021), ULK1 inhibition (SBI, dose-dependently) or silencing significantly blocked conidial uptake (Figures 4K,L). CR3 loss reduced internalization, whereas CR3 overexpression had no effect (Figure 4M). Together, ULK1 and CR3 play important roles in modulating inflammatory factor release and conidial internalization in Beas2B cells during *A. fumigatus* infection.

## Discussion

*Aspergillus fumigatus* is a major fungal pathogen causing invasive aspergillosis, particularly in vulnerable hosts (Margalit et al., 2021). While the role of autophagy in immune cells against *A. fumigatus* is established, its function in epithelial cells is less defined (Li et al., 2020). This study investigated autophagy in Beas2B cells following *A. fumigatus* internalization. We identified a novel, ULK1-dominated, LAP-like autophagic pathway crucial for autophagosome formation. This process was time- and dose-dependent, showed LC3-II conversion, and was independent of Dectin-1. Importantly, CR3 deficiency enhanced autophagy, suggesting that CR3 signaling negatively regulates this process via the AMPK/ULK1 pathway. These findings clarify epithelial autophagic responses to fungi and may offer new therapeutic targets.

Canonical autophagy primarily targets damaged or excessive intracellular proteins and organelles for degradation and recycling, whereas LAP represents a selective autophagic process specifically directed toward extracellular material, such as pathogens or cellular debris (Mizushima and Komatsu, 2011). Mechanistically, LAP involves the direct conjugation of LC3 to phagosomal membranes, forming LC3-positive phagolysosomes, a process independent of the canonical ULK1/Atg13 autophagy initiation complex. LAP provides an efficient pathway for cells to eliminate foreign substances and maintain homeostasis, which is critical for combating pathogen infections and preserving tissue integrity (Peña-Martinez et al., 2022). Our findings regarding the interconnection between these two autophagic modalities suggest that the observed phenomenon may represent a manifestation of LAP. In macrophages, LAP depends on Rubicon and NOX2-mediated ROS generation to form single-membrane autophagosomes that rapidly fuse with lysosomes (Masud et al., 2019). However, we identified that in Beas2B cells, although the autophagosomes exhibit single-membrane morphology, they rely on ULK1 rather than Rubicon, indicating the potential existence of a “hybrid” autophagic mechanism. This process shares morphological similarities with LAP but aligns more closely with the molecular machinery of canonical autophagy.

Rubicon, a pivotal regulator in the LAP process that is essential for the stable association of lipidated LC3-II with phagocytic vesicles (Martinez et al., 2015), showed no significant changes until 10 h post-infection, with the exception of a slight downward trend observed in the later stages. More notably, ULK1, a crucial protein within the initiation complex of canonical autophagy, exhibited a marked increase in both expression and phosphorylation at serine residues 555 and 757. Consistent with these observations, the knockdown of ULK1, but not Rubicon, largely inhibited the conversion to LC3-II in Beas2B cells during *A.fumigatus* infection. In the canonical autophagy pathway, the ULK1 complex is activated during the initial stages of autophagosome formation, facilitating the nucleation of autophagic precursors (Hwang et al., 2017). In the LAP process, ULK1 may also play a role in the nucleation or maturation of autophagosomes, although this role may not be as critical since LAP nucleation primarily depends on phagocytic events (Jannat et al., 2019). Nonetheless, our findings offer the first evidence that *A. fumigatus* conidia are internalized by epithelial cells and trigger autophagy via the ULK1 complex. This represents a novel regulatory pathway in the interaction between pathogens and host cells.

It was intriguing to find out that CR3 receptor strongly and negatively dominates both basal and *A. fumigatus* conidia-induced LC3-II conversion in epithelial cells. CR3 deficiency led to a significant increase in LC3-II; in contrast, its overexpression inhibited LC3-II conversion, suggesting that CR3 acts as a negative regulator of LC3-II formation in epithelial cells. This novel negative regulatory mechanism is of particular interesting. CR3, also known as Mac-1 or integrin αM*β*2, is a classical member of the integrin family that recognizes diverse microbial components. Previous reports indicate that LAP initiated by the ITGAM-ITGB2/Mac-1 complex enhances immunity to *Listeria monocytogenes* (Herb et al., 2018) and that CR3 facilitates efficient phagocytosis and elimination of this bacterium by inducing LAP (Gluschko et al., 2018). Another study demonstrated that CR3 is expressed on the surface of human alveolar epithelial cells, where it recognizes β-1,3-glucan to mediate *A. fumigatus* internalization by increasing intracellular phosphatidic acid via FAK activation (Han et al., 2021). The loss of CR3 may trigger mechanisms that promote the autophagy pathway. Indeed, as shown in Figure 3, the phosphorylation of ULK1 at Ser555 and Ser757 was upregulated upon CR3 loss and unaffected by CR3 overexpression, revealing a non-canonical inhibitory role for CR3 in autophagy regulation.

Additionally, CR3 deletion is accompanied by AMPK upregulation, and AMPK-mediated phosphorylation of ULK1 is a characteristic feature of canonical autophagy (Wang et al., 2024). mTORC1 inhibits the initiation of autophagy by phosphorylating ULK1 and Atg13 within the ULK1 complex, as well as Atg14 in the VPS34 complex, thereby reducing the enzymatic activity of ULK1 and VPS34 and suppressing autophagosome formation (Yuan et al., 2013; Kim et al., 2011). Therefore, CR3 signaling may activate mTORC1 through the integrin-Akt pathway, with constitutive CR3 signaling possibly maintaining mTORC1 activity, inhibiting AMPK and ULK1, and thus suppressing autophagy. The absence of CR3 relieves mTORC1 inhibition of AMPK, leading to AMPK phosphorylation of ULK1 and the promotion of autophagosome formation. ULK1 is an initiating factor of canonical autophagy, and its upregulation is consistent with AMPK activation, suggesting that AMPK promotes autophagy by phosphorylating ULK1 at Ser317. This supports the notion that the deletion of CR3 activates the autophagy regulatory pathway through the AMPK/ULK1 axis, thereby enhancing autophagy. This regulatory mechanism is biologically significant. In the early stages of infection, CR3 may help prevent excessive inflammatory damage by limiting autophagy. However, chronic suppression of autophagy could undermine the ability to clear fungi, potentially leading to the progression of the infection.

Further, we investigated which component of *A. fumigatus* triggers this ULK1-dominated LC3-II conversion. Surprisingly, as the putative PAMPs, such as the classical polysaccharides on the conidial cell wall including *β*-1,3-glucan, mannan, zymosan and laminarin, did not induce the LC3-II conversion when used to stimulate epithelial cells at various time points or concentrations. These results differ significantly from LAP and many innate immune signal pathways in myeloid cells, which are predominantly triggered by the polysaccharides on the conidial surface (Ahmad et al., 2024). Moreover, the deletion of neither the hydrophobins RodA and RodB nor pksP (a gene encoding an enzyme for melanin synthesis) affected the conidia-induced LC3-II conversion. It has been reported that *A. fumigatus* melanin can sequester intracellular Ca^2+^ and inhibit calcium-calmodulin signaling to prevent LC3-associated phagocytosis (Kyrmizi et al., 2018). In contrast, melanin had little effect on the autophagy process in Beas2B cells during *A. fumigatus* internalization. This may be because polysaccharides on the conidial surface are obscured (e.g., by a hydrophobic protein layer) and cannot be recognized by pattern recognition receptors (PRRs). Consistent with the non-involvement of *β*-1,3-glucan in inducing the autophagy in epithelial cells, Dectin-1, a critical receptor for fungus-host interactions, did not participate in regulating LC3-II elevation. This suggests that airway epithelial cells may recognize conidia through atypical receptors (such as TLRs or mechanosensory receptors) (Lehmann et al., 2018) or activate autophagy via intracellular sensors (such as NLRs) after internalization (Bogefors et al., 2010). Additionally, heat-killed *A. fumigatus* conidia lost the ability to induce the LC3-II conversion, suggesting that some heat-sensitive components, such as proteins, might dominate the induction of LC3-II conversion in Beas2B cells. Further investigation is necessary to explore the potential molecules on the conidial surface responsible for inducing the autophagy in epithelial cells.

This study primarily utilized Beas2B as a model to simulate the early process of *A. fumigatus* conidial internalization. The key findings obtained in A549 cells were largely consistent with those in Beas2B cells, supporting the robustness of our observations. However, subtle yet clear differences were also noted between these two cell lines. Notably, the effects induced by *A. fumigatus* infection, including autophagy-related responses and molecular changes, were more pronounced in Beas2B cells compared to A549 cells. Although CD11b expression levels are comparable between Beas2B and A549 cells, the autophagic responses induced by *A. fumigatus* infection are much more prominent in Beas2B cells. These observations suggest that the CD11b/CR3-regulated LAP-like pathway we identified is cell-context dependent, and the full activation of this pathway requires a specific cellular background that is more evident in Beas2B cells. Therefore, while A549 cells can serve as a complementary cell model in future studies, Beas2B cells appear to be more sensitive and suitable for investigating host responses against *A. fumigatus* infection within our experimental system. It should be acknowledged, however, that airway epithelial cells lack the complex recognition and response mechanisms of professional immune cells, such as macrophages and dendritic cells. Therefore, whether the autophagy regulatory mechanisms mediated by ULK1 and CR3 are specific to immune cell types requires further validation. The key receptors involved in conidial-induced autophagy have not yet been identified. Future research will need to employ targeted screening or omics technologies to elucidate these recognition mechanisms. Additionally, it is essential to investigate whether different fungal pathogens, such as Candida and Cryptococcus, share similar pathways. This is crucial for understanding their global role in the host’s antifungal immune response.

**Figure 5:**
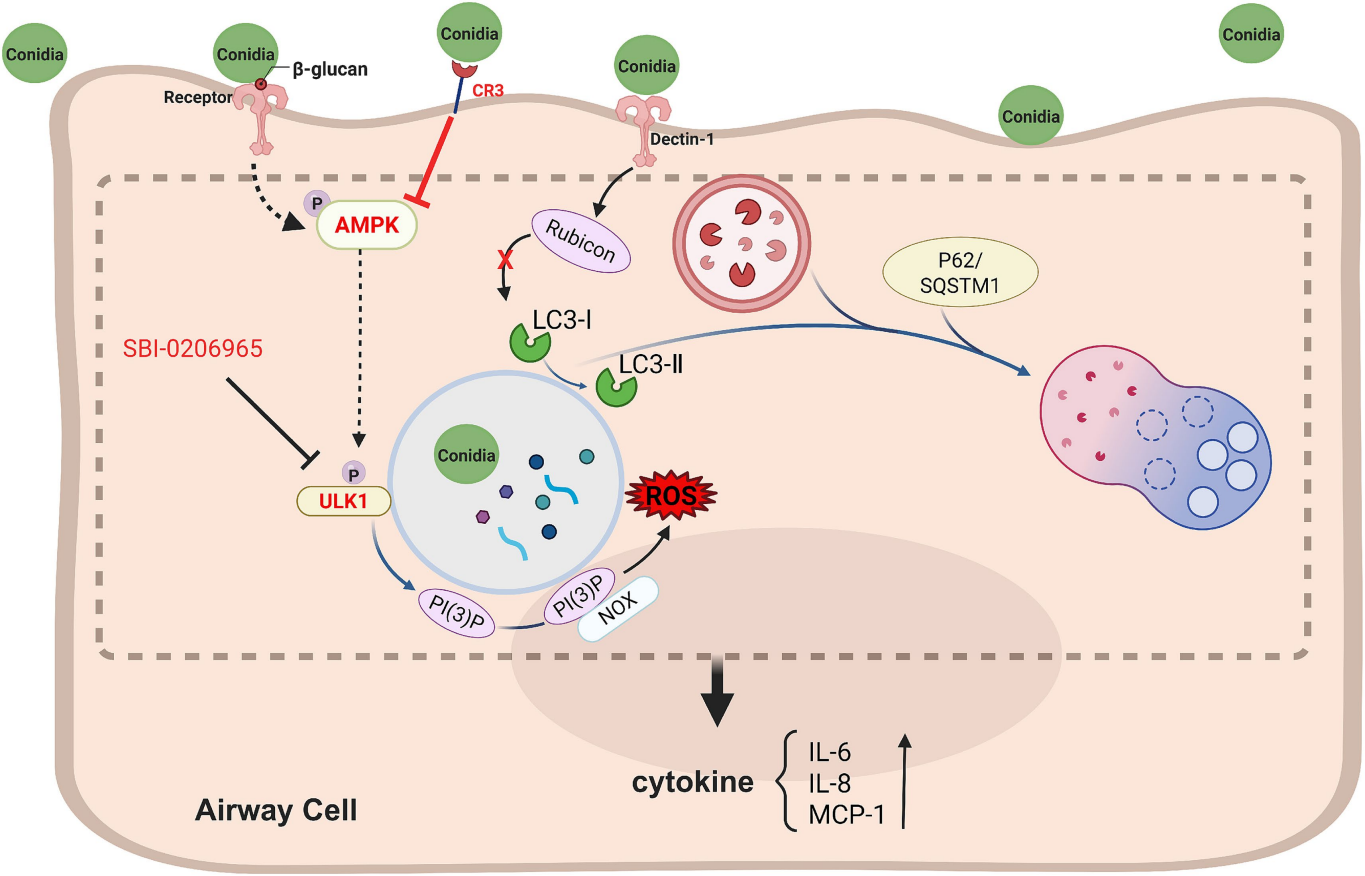
Model of a novel ULK1-mediated LAP-like pathway in airway cells during *A. fumigatus* conidial internalization. During the interaction between *A. fumigatus* conidia and airway cells, several changes occur: intracellular LC3-II levels, phosphorylation of AMPK and ULK1, and reactive oxygen species (ROS) content all increase. Additionally, the release of cytokines—including IL-6, IL-8, and MCP-1—also rises. Inhibiting ULK1 activity or silencing its expression can suppress the increase in LC3-II levels and cytokine release triggered by *A. fumigatus*. Notably, common fungal polysaccharides and Dectin-1 did not impact this process, but the loss of complement receptor 3 elevated both basal and conidia-induced autophagy, correlating with increased AMPK expression. Dashed lines indicate signal pathway interactions hypothesized to occur in airway cells.

In summary, our findings unveil a novel ULK1-mediated LAP-like pathway in Beas2B cells during *A. fumigatus* conidial internalization. This process is time- and dose-dependent, independent of classical pattern recognition receptors, and enhanced by CR3 deficiency, which is linked to AMPK upregulation (Figure 5). This study illuminates a previously unrecognized mechanism of epithelial cell autophagy induction by fungal pathogens, suggesting potential therapeutic strategies for treating invasive aspergillosis.

## Data Availability

The original contributions presented in the study are included in the article/Supplementary material, further inquiries can be directed to the corresponding author.

## Supplementary material

SUPPLEMENTARY FIGURE S1 Increased LC3-II expression and monolayer membrane-coated autophagosome formation in A549 cells following conidia infection. **(A)** Expression of LC3-I and LC3-II in A549 following infected by B5233 wild-type live conidia at 0, 2, 4, 6, 8, and 10 h. **(B)** Relative expression levels of LC3-I and LC3-II at the various time points. **(C)** Fluorescence microscopy images of LC3B (red), *A. fumigatus* ATCC13073 conidia (constitutively expressing green fluorescent protein), and DAPI staining (blue) in A549 cells following infected by conidia at 0, 4, 6, and 8 h. **(D)** Electron microscopy images of cellular structural changes over time at 2, 4, 8, and 12 h. The magnification progressively increases from left to right, with each subsequent column of images depicting the area enclosed within the red box of the preceding image. The red arrow in each image points to the monolayer membrane encapsulating the *A. fumigatus* spores. **(E-F)** Expression levels of LC3-I and LC3-II in A549 cells with CR3 knockdown (*CR3^-/-^*) or overexpression (*CR3OE*) after internalization by conidia. ^*^*p* < 0.05; ^**^*p* < 0.01.

SUPPLEMENTARY FIGURE S2 Autophagy in Beas2B is triggered by *A. fumigatus* conidia via heat-sensitive elements, not by β-glucan, melanin, or surface polysaccharides. **(A–D)** Expression levels of LC3-I and LC3-II in Beas2B treated with 100μg/ml β-1,3-glucan, manan, zymosan, and laminarin at various time points (0, 15, 30, 60, 120, and 240 min). **(E)** Expression levels of LC3-I and LC3-II in Beas2B infected with B5233 wild-type live conidia different mutants after 6 h. **(F)** Expression levels of LC3-I and LC3-II in Beas2B following infection with heat-killed B5233 wild type conidia at different time intervals (0, 2, 4, 6, 8, 10, and 12 h).

SUPPLEMENTARY FIGURE S3 Rubicon and dectin-1 are not key regulators in *A. fumigatus* conidia-induced autophagy in Beas2B. **(A)** Expression levels of Rubicon in Beas2B after infected by B5233 wild-type live conidia at 0, 2, 4, 6, 8, and 10 h. **(B–D)** Quantification of expression levels of Rubicon, Atg5, and VPS34 in Beas2B infected by conidia at 0, 2, 4, 6, 8, and 10 h. **(E)** Expression levels of p62 in Beas2B after infected by conidia at 0, 2, 4, 6, 8, 10, and 12 h. **(F)** Quantification of expression levels of p62 in Beas2B infected by conidia at 0, 2, 4, 6, 8, 10, and 12 h. **(G)** ROS levels of Beas2B after infected by conidia at 0, 2, 4, 6, 8, 10, and 12 h. **(H-I)** Expression levels of LC3-I and LC3-II in Beas2B following internalization of live conidia, with Rubicon-siRNA or VPS34-siRNA. **(J)** Expression levels of Dectin-1 in Beas2B after infected by conidia at 0, 2, 4, 6, 8, and 10 h. **(K)** Expression levels of LC3-I and LC3-II in Dectin-1^-/-^ and Dectin-1 OE Beas2B after infected by conidia.

SUPPLEMENTARY FIGURE S4 mRNA expression of Atg5, VPS34, Rubicon, ULK1, and CD11b during *Aspergillus fumigatus* infection in Beas2B and A549 cells. mRNA expression levels of Atg5, VPS34, Rubicon, ULK1, and CD11b in Beas2B and A549 cells after infected by B5233 wild-type live conidia (MOI=10) at 0, 1, 2, 4, 6, and 8 h. ^*^*p* < 0.05.
