# Supplementary figures and images for: ULK1 mediated autophagy in airway cells during Aspergillus infection
Source: Front Microbiol. 2026 Mar 17;17:1756294. doi: 10.3389/fmicb.2026.1756294 (PMC13036178; doi:10.3389/fmicb.2026.1756294)

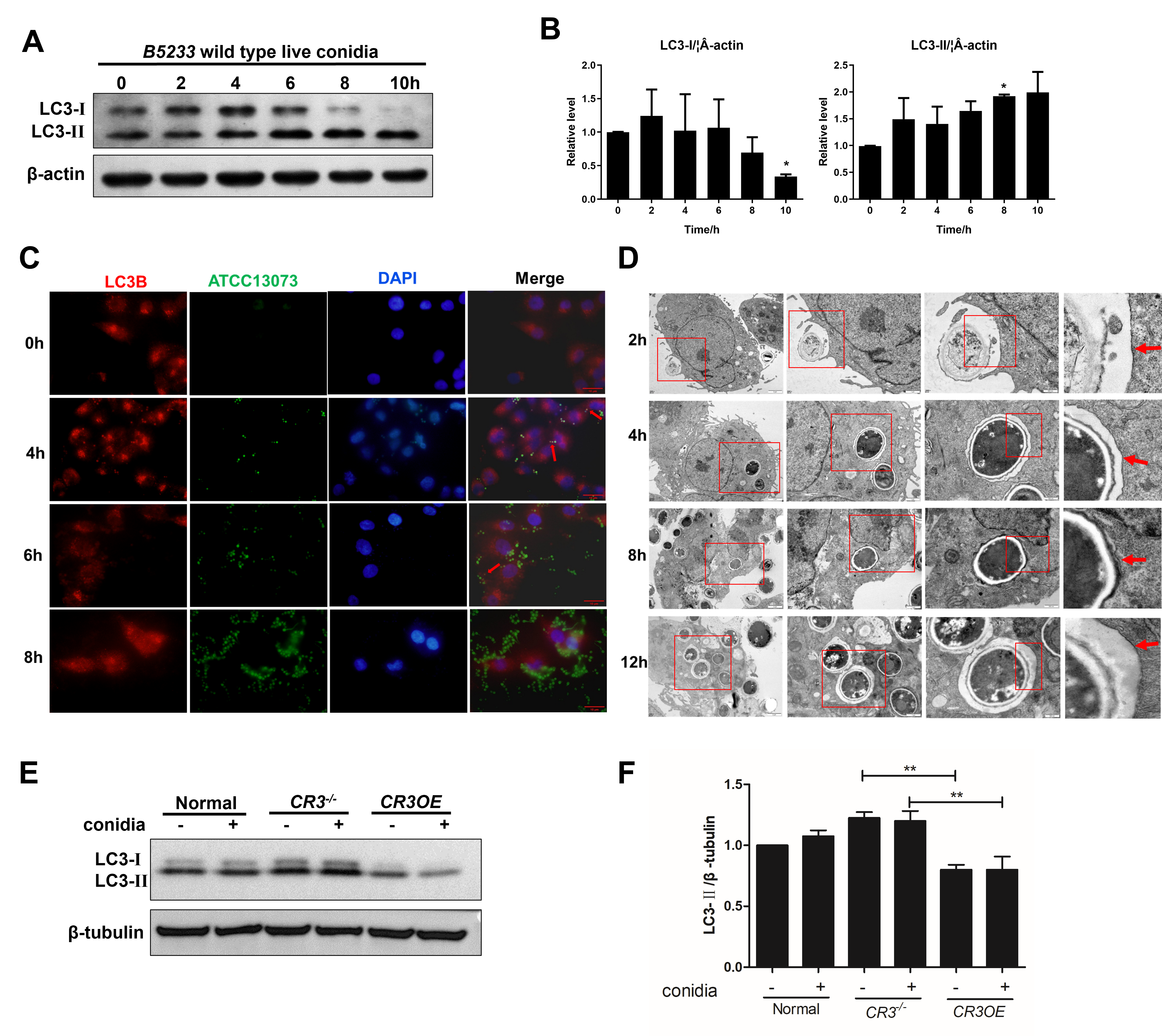

Supplement: SUPPLEMENTARY FIGURE S1 — Increased LC3-II expression and monolayer membrane-coated autophagosome formation in A549 cells following conidia infection. (A) Expression of LC3-I and LC3-II in A549 following infected by B5233 wild-type live conidia at 0, 2, 4, 6, 8, and 10 h. (B) Relative expression levels of LC3-I and LC3-II at the various time points. (C) Fluorescence microscopy images of LC3B (red), A. fumigatus ATCC13073 conidia (constitutively expressing green fluorescent protein), and DAPI staining (blue) in A549 cells following infected by conidia at 0, 4, 6, and 8 h. (D) Electron microscopy images of cellular structural changes over time at 2, 4, 8, and 12 h. The magnification progressively increases from left to right, with each subsequent column of images depicting the area enclosed within the red box of the preceding image. The red arrow in each image points to the monolayer membrane encapsulating the A. fumigatus spores. (E-F) Expression levels of LC3-I and LC3-II in A549 cells with CR3 knockdown (CR3-/-) or overexpression (CR3OE) after internalization by conidia. *p < 0.05; **p < 0.01. [file Image_1.TIF]

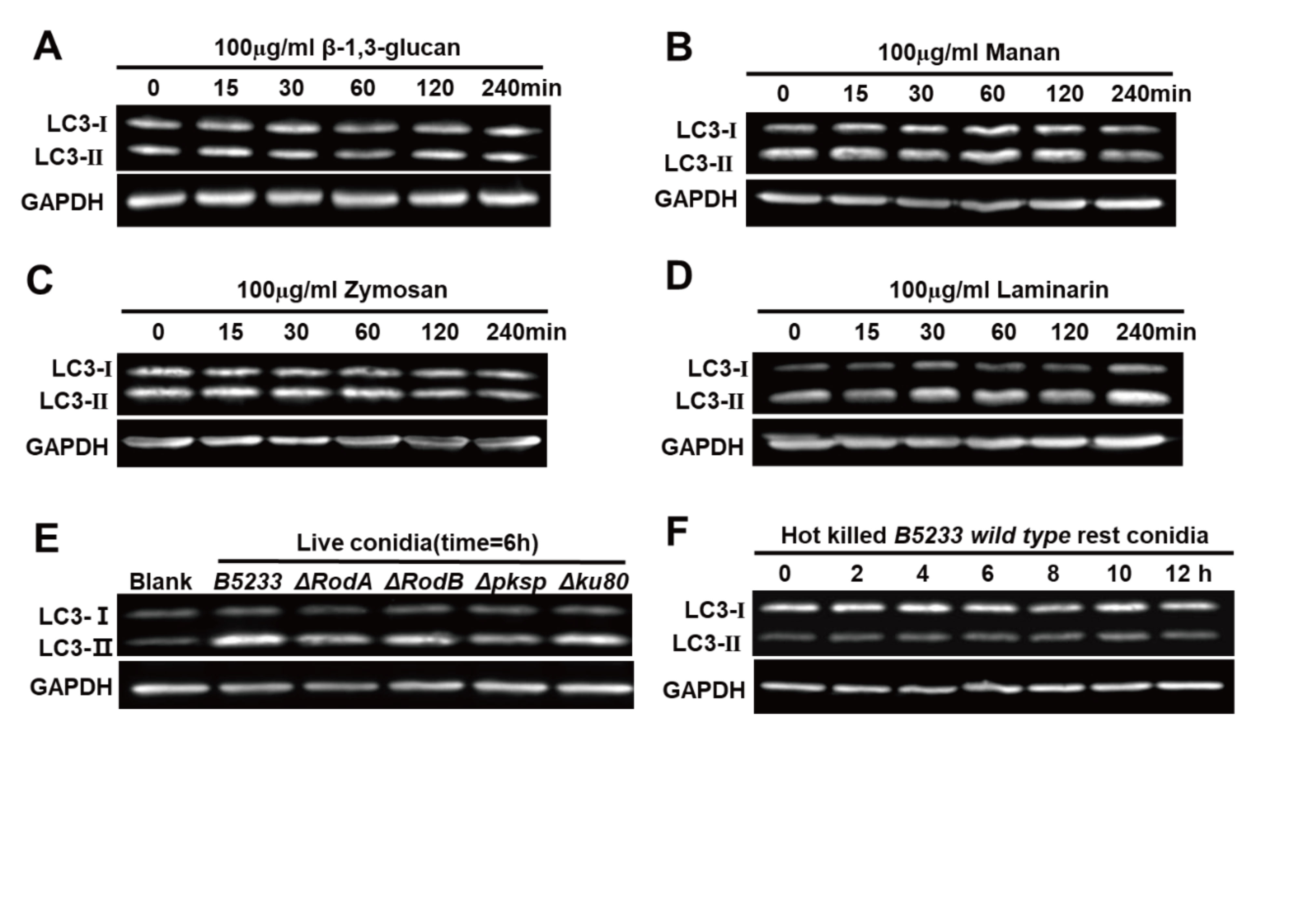

Supplement: SUPPLEMENTARY FIGURE S2 — Autophagy in Beas2B is triggered by A. fumigatus conidia via heat-sensitive elements, not by β-glucan, melanin, or surface polysaccharides. (A–D) Expression levels of LC3-I and LC3-II in Beas2B treated with 100μg/ml β-1,3-glucan, manan, zymosan, and laminarin at various time points (0, 15, 30, 60, 120, and 240 min). (E) Expression levels of LC3-I and LC3-II in Beas2B infected with B5233 wild-type live conidia different mutants after 6 h. (F) Expression levels of LC3-I and LC3-II in Beas2B following infection with heat-killed B5233 wild type conidia at different time intervals (0, 2, 4, 6, 8, 10, and 12 h). [file Image_2.JPEG]

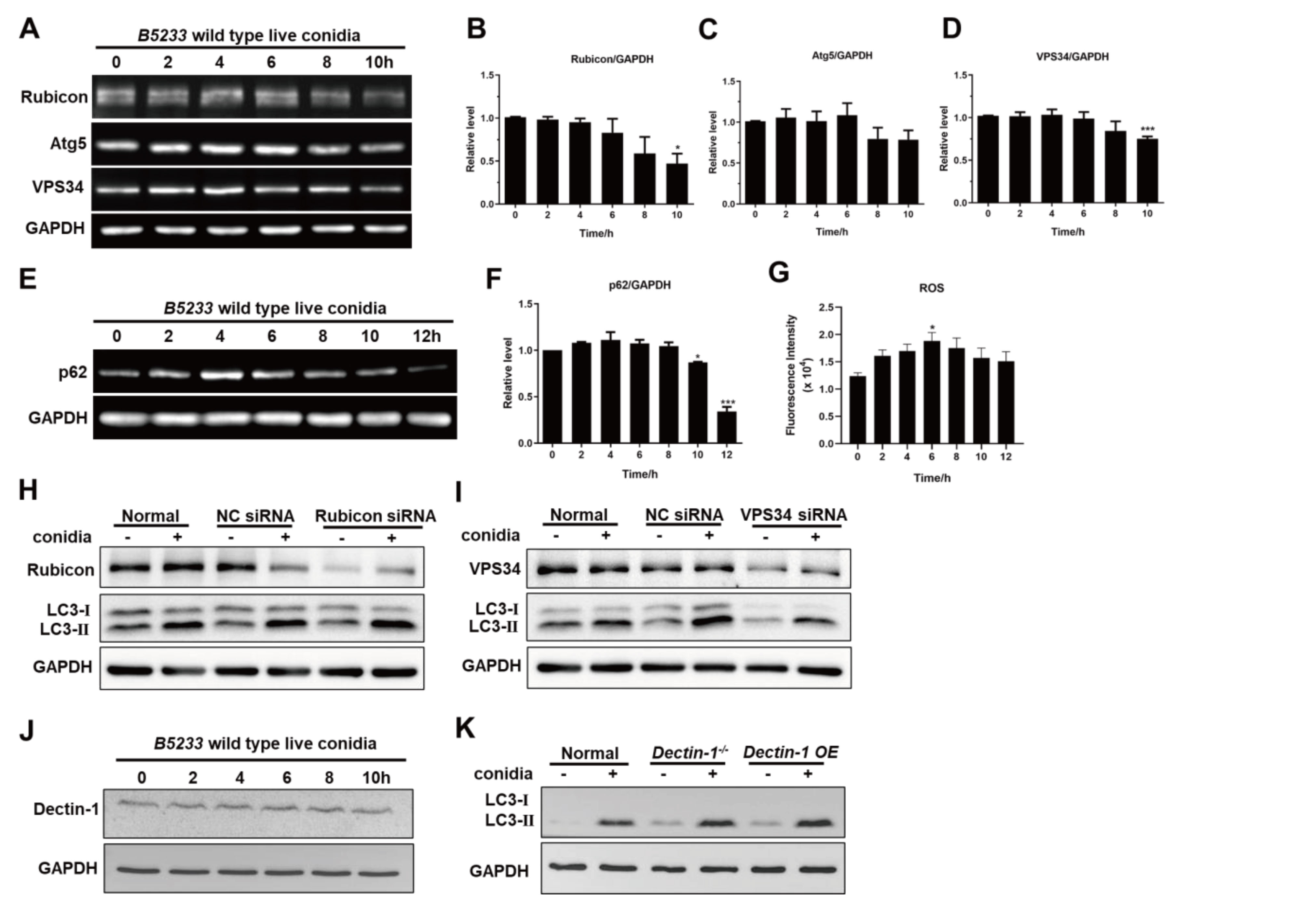

Supplement: SUPPLEMENTARY FIGURE S3 — Rubicon and dectin-1 are not key regulators in A. fumigatus conidia-induced autophagy in Beas2B. (A) Expression levels of Rubicon in Beas2B after infected by B5233 wild-type live conidia at 0, 2, 4, 6, 8, and 10 h. (B–D) Quantification of expression levels of Rubicon, Atg5, and VPS34 in Beas2B infected by conidia at 0, 2, 4, 6, 8, and 10 h. (E) Expression levels of p62 in Beas2B after infected by conidia at 0, 2, 4, 6, 8, 10, and 12 h. (F) Quantification of expression levels of p62 in Beas2B infected by conidia at 0, 2, 4, 6, 8, 10, and 12 h. (G) ROS levels of Beas2B after infected by conidia at 0, 2, 4, 6, 8, 10, and 12 h. (H-I) Expression levels of LC3-I and LC3-II in Beas2B following internalization of live conidia, with Rubicon-siRNA or VPS34-siRNA. (J) Expression levels of Dectin-1 in Beas2B after infected by conidia at 0, 2, 4, 6, 8, and 10 h. (K) Expression levels of LC3-I and LC3-II in Dectin-1-/- and Dectin-1 OE Beas2B after infected by conidia. [file Image_3.JPEG]

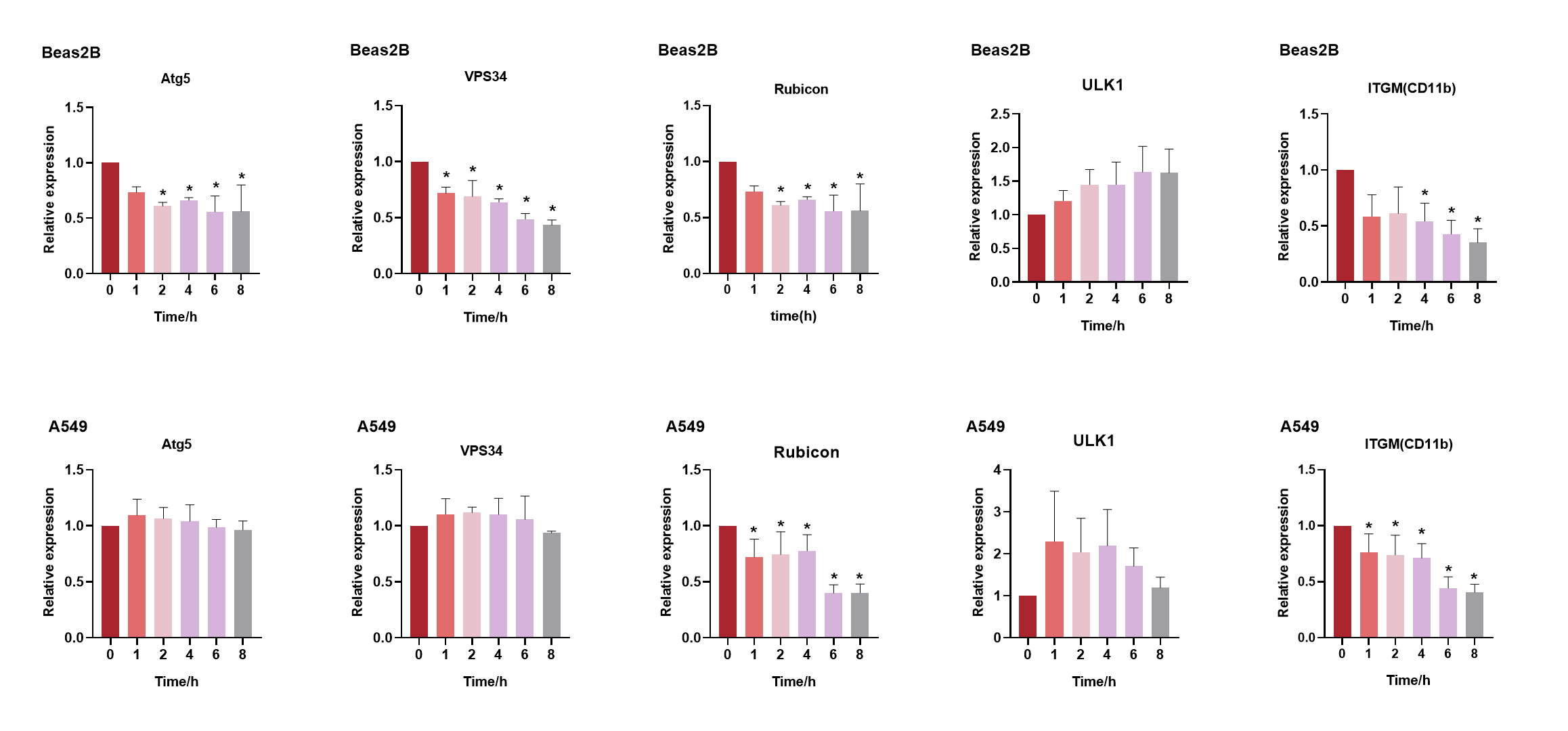

Supplement: SUPPLEMENTARY FIGURE S4 — mRNA expression of Atg5, VPS34, Rubicon, ULK1, and CD11b during Aspergillus fumigatus infection in Beas2B and A549 cells. mRNA expression levels of Atg5, VPS34, Rubicon, ULK1, and CD11b in Beas2B and A549 cells after infected by B5233 wild-type live conidia (MOI=10) at 0, 1, 2, 4, 6, and 8 h. *p < 0.05. [file Image_4.TIF]
